# Complete Nucleotide Sequence, Genome Organization, and Comparative Genomic Analyses of Citrus Yellow-Vein Associated Virus (CYVaV)

**DOI:** 10.3389/fmicb.2021.683130

**Published:** 2021-06-08

**Authors:** Sun-Jung Kwon, Sohrab Bodaghi, Tyler Dang, Kiran R. Gadhave, Thien Ho, Fatima Osman, Maher Al Rwahnih, Ioannis E. Tzanetakis, Anne E. Simon, Georgios Vidalakis

**Affiliations:** ^1^Department of Microbiology and Plant Pathology, University of California, Riverside, Riverside, CA, United States; ^2^Institutes of Green Bio Science and Technology, Seoul National University, Pyeongchang, South Korea; ^3^Department of Entomology and Plant Pathology, University of Arkansas, Fayetteville, AR, United States; ^4^Department of Plant Pathology, University of California, Davis, Davis, CA, United States; ^5^Department of Cell Biology and Molecular Genetics, University of Maryland, College Park, MD, United States

**Keywords:** *Tombusviridae*, bio-indexing, plant virus characterization, virus-virus interactions, high-throughput sequencing, VirFind, small RNA virus

## Abstract

Citrus yellow-vein disease (CYVD) was first reported in California in 1957. We now report that CYVD is associated with a virus-like agent, provisionally named citrus yellow-vein associated virus (CYVaV). The CYVaV RNA genome has 2,692 nucleotides and codes for two discernable open reading frames (ORFs). ORF1 encodes a protein of 190 amino acid (aa) whereas ORF2 is presumably generated by a −1 ribosomal frameshifting event just upstream of the ORF1 termination signal. The frameshift product (717 aa) encodes the RNA-dependent RNA polymerase (RdRp). Phylogenetic analyses suggest that CYVaV is closely related to unclassified virus-like RNAs in the family *Tombusviridae*. Bio-indexing and RNA-seq experiments indicate that CYVaV can induce yellow vein symptoms independently of known citrus viruses or viroids.

## Introduction

Subviral RNAs are one of the smallest plant pathogens (Shimura and Masuta, 2016). They vary in length, complexity, and functions, in that they rely on helper viruses for at least one of the functions: replication, encapsidation, or *in planta* movement. For instance, non-coding satellite RNAs are incapable of all three functions (Badar et al., 2020), translated RNAs are capable of encapsidation, but lack the ability to replicate and move in plants (Gnanasekaran and Chakraborty, 2018), whereas coat protein-dependent RNA replicons are capable of synthesizing RNA-dependent RNA polymerase (RdRp) but rely on helper viruses for at least encapsidation (Campbell et al., 2020). The association of umbraviruses with various viruses in the family *Luteoviridae* has been extensively studied (Taliansky and Robinson, 2003). Umbraviruses typically rely on the coat protein of a helper virus, for both encapsidation and transmission.

**Figure 1:**
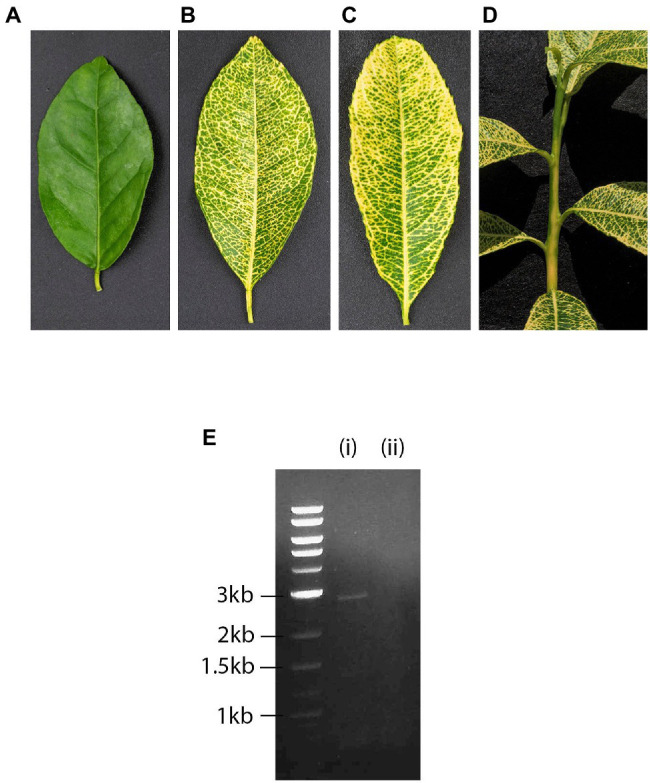
Healthy **(A)** and yellow-vein infected leaf of citrus indicator Mexican lime *Citrus aurantifolia* (Christm.) Swingle **(B)**. Yellow-vein infected leaf **(C)** and stem **(D)** of citrus indicator “Etrog” citron (*C. medica* L.). Agarose gel electrophoresis of double-stranded RNAs (dsRNAs) extracted from (i) “Etrog” citron graft-inoculated with yellow-vein isolate YV-920C, expressing yellow vein symptoms and (ii) healthy control **(E)**. Size markers are shown to the left of the gel.

In 1969, a citrus yellow-vein disease (CYVD) accession was introduced by Dr. L. G. Weathers most likely from Tulare County, California, into the disease bank of the Citrus Clonal Protection Program (CCPP) at the University of California, Riverside (UCR) under the name YV-920. The original 1969 YV-920 isolate was used in a series of bio-indexing experiments on various citrus indicators, resulting in consistent expression of yellow vein symptoms while testing negative for other graft-transmissible diseases of citrus, such as exocortis, psorosis, tristeza, and concave gum (CCPP bio-indexing records). In a bio-indexing experiment in September 1985, an “Etrog” citron (*Citrus medica* L.) tree was graft-inoculated with YV-920, and upon expressing yellow vein symptoms it was held at the CCPP as the CYVD source YV-920C. Since the first report of CYDV in Eustis limequat [*Citrus aurantifolia* (Christm.) Swing. x *Fortunella japonica* (Thunb.) Swing.] in California by Weathers (1957), there have been extensive studies on transmission, host range, and symptom development (Weathers, 1960, 1961). Unlike other citrus virus and viroid diseases, CYDV is uncommon and is not transmissible either mechanically, *via* dodder or by arthropods. It is readily graft-transmissible to multiple citrus species with the exception of *Poncirus trifoliata* (L.) Raf. Sensitive indicators such as “Etrog” citron (*C. medica* L.) and Mexican lime [*C. aurantifolia* (Christm.) Swingle] develop bright yellow discoloration of petioles and veins. In some cases, the symptoms extend into the young flushes and stems. The yellowing is persistent and visible in older leaves and branches (Figures 1A–D).

An important attribute of the CYVD pathogen is its ability to interact with other graft-transmissible agents resulting in either suppression or enhancement of symptoms. Plants infected with the CYVD pathogen and citrus psorosis virus (CPsV, *Ophiovirus*, *Aspiviridae*) showed pronounced psorosis and suppressed yellow vein symptoms. Weathers noted that CYVD “… is caused by a virus or viruses…” that have “…some type of interaction with the unrelated viruses of psorosis and vein enation.” Most importantly, however, Weathers indicated that CYVD “… is different from any previously described diseases of citrus” (Weathers, 1957, 1960). Contrary to psorosis, co-inoculation of indicators with the CYVD pathogen and citrus vein enation virus (CVEV, *Enamovirus*, *Luteoviridae*) enhanced yellow vein symptoms and caused stunting and occasional plant death (Weathers, 1960, 1961, 1963; Vives et al., 2013; Moreno et al., 2015).

Here, we provide evidence that the CYVD associated pathogen is a novel 2,692 nt citrus yellow-vein associated virus (CYVaV), which is capable of inducing yellow vein symptoms independently of other known citrus viruses or viroids.

## Materials and Methods

### Plant Materials

Citrus plants (“Etrog” citron, sour orange and Mexican lime) used in this study were graft-inoculated with YV-920C as original source of inoculum and maintained in the CCPP quarantine greenhouse at 22–29°C and 16 h light regime.

### Double-Stranded RNA Extraction

Stems with yellow vein symptomatic leaves were harvested 2.5 years after inoculation, and double-stranded RNA (dsRNA) was extracted from 20 g of bark scrapings using two-cycles of CF11 column chromatography as previously described (Valverde and Nameth, 1990; Kwon et al., 2007). dsRNA was enriched after two cycles of 3 volume 95% ethanol, 3 M sodium acetate (pH 5.5) precipitations, and eventually the pellet was resuspended in 125 μl of sterile water. Enriched dsRNA was subjected to electrophoresis through 1% agarose gels, and nucleic acids were visualized by ethidium bromide staining.

### Cloning and Sequencing

The enriched dsRNA preparation was denatured in 40% DMSO at 95°C for 10 min. The Superscript™ choice system (Invitrogen, Thermo Fisher Scientific, Waltham, MA, United States) was used to construct a cDNA library using random primers according to the manufacturer’s instructions. *Taq* DNA polymerase was used to add adenylate residues to the 3' ends of the double-stranded cDNAs, which were then ligated into pGEM-T easy vector (Promega, Madison, WI, United States). Recombinant colonies were randomly selected, and recombinant plasmids were Sanger-sequenced at the UC Riverside Genomics Core Facility. The sequences of 48 cDNA clones were assembled using the ContigExpress tool (Vector NTI Advance 10-InforMax) and compared with the NCBI nucleotide and protein databases. Two assembled contigs with non-contiguous sequences (930 and 1,673 nt) with a low *E*-value to viruses were obtained by BLAST analysis. The sequence gap between the two contigs was filled by real-time polymerase chain reaction (RT-PCR) with specific primers based on sequences flanking the gap (Supplementary Table 1). The sequence gap was subsequently determined and one large contig of 2,642 nt was assembled. The ends were amplified by RACE-PCR, cloned, and sequenced, as previously described (Kwon et al., 2014). Potential open reading frames (ORFs) and conserved domain(s) were determined using the NCBI ORF finder and CD-search utilities, respectively (Marchler-Bauer and Bryant, 2004; Marchler-Bauer et al., 2009). The CYVaV sequence was deposited in GenBank under the accession number JX101610.

### RT-PCR

Two sets of CYVaV specific RT-PCR primers were designed at the 5' (F1_49-69_, 5'-CCA GAC AGG TGT TTC GAG CAT-3'; R1_566-584_, 5'-CAA TCA CTG CAA ATC GCG A-3', 536 nt amplicon) and 3' end (F5_2117-2138_, 5'-GAA CAC GGA AGT GAG TGG TAC G-3'; R5_2608-2633_, 5'-AGA ATG CTA CTC TGA GTA CAA GCC C-3', 517-nt amplicon) of the JX101610 sequence. Reactions were performed with the OneStep RT-PCR Kit (QIAGEN, Valencia, CA, United States) with the same final concentration of buffers and primers (6 μM) per manufacturer’s instructions. The RT step was carried out at 50°C for 30 min followed by a 15 min denaturation at 95°C for activation of the hot-start *Taq* polymerase. The PCR conditions were as follows, 35 cycles at 94°C for 30 s, 56°C for 30 s, 72°C for 1 min, and a final extension step at 72°C for 10 min. PCR products were analyzed by electrophoresis on 1.5% agarose gels buffered in TAE (0.04 M Tris–acetate, 1 mM EDTA, pH 8) and visualized by UV light after ethidium bromide staining and sequenced at the UCR Genomics Core Facility.

### Sequence and Phylogenetic Analyses

Citrus yellow-vein associated virus sequence identities were analyzed by comparing with sequences in GenBank using BLASTn and BLASTx. Nucleotide and amino acid sequences retrieved from GenBank were aligned using CLUSTAL W (Thompson et al., 1994). Illustration of the conserved RdRp aa sequences and motifs I-VIII (Koonin, 1991) was prepared using GeneDoc v2.7.00 (Nicholas and Nicholas, 1997). The 5' and 3' secondary structures were analyzed using phylogenetic comparisons, determining if structures similar to known ones in the *Tombusviridae* can be formed using the complement searching function in the RNA structure drawing program RNA2Drawer (https://rna2drawer.app/; Johnson et al., 2019). The conserved RdRp aa sequences were used to construct a Maximum likelihood phylogenetic tree on MEGA X using the Jones-Taylor-Thornton substitution model (Kumar et al., 2018). Comparative analysis on the RdRp aa sequences with orthologs from phylogenetically related viruses and unclassified virus-like RNAs in the family *Tombusviridae* was performed using Megalign (Lasergene, DNAstar, Madison, WI, United States). A Maximum likelihood phylogenetic tree for the nucleotide sequence of CYVaV-related viruses and virus-like RNAs was constructed using the Tamura-Nei substitution model for complete nucleotide sequence with rates among sites gamma distributed with invariant site (G + I). Bootstrap values were calculated using 1,000 pseudo replications. Clades with less than 70% bootstrap support were collapsed.

### Vein Enation Biological Indexing and RNA-seq

Three serial passage bio-indexing experiments were conducted in 2009, 2016, and 2020. The occurrence of vein enation and yellow vein was recorded. About 3–10 “Etrog” citron (yellow vein indicator), sour orange (*C. aurantium* L., vein enation indicator), and Mexican lime (yellow vein and vein enation indicator) were used per experiment. The 2009 (experiment #3163) graft-transmission experiment used the YV-920C as the source of inoculum. In the 2016 experiment (experiment #3358), a 2009 yellow vein symptomatic “Etrog” citron plant (experiment #3163‐ tree 02) was used as the source of inoculum. In the 2020 experiment, a 2016 Mexican lime plant (experiment #3358‐ tree 70) expressing only yellow vein symptoms was used as the source of inoculum. Non-inoculated indicators and vein enation controls (CCPP Disease Bank isolates VE 701 and 709) were included in all experiments. The indicators were maintained under cool greenhouse conditions (24–27°C) and observed periodically for symptoms expression (Roistacher, 1991; Vidalakis et al., 2004).

RNA-seq was performed to determine the viral compositions of the 2009 and 2016 serial passage bio-indexing experiments. A series of non-inoculated and positive controls from the CCPP Lindcove Foundation Facility (Exeter, CA) and the CCPP Disease Bank at the Rubidoux Quarantine Facility (Riverside, CA), respectively, were also tested. Libraries were constructed using the TruSeq Stranded Total RNA Sample Library Prep with plant rRNA depletion (Illumina, San Diego, CA, United States) using TRIazol® (Thermo Fisher Scientific, Waltham, MA, United States) -extracted RNA. The libraries were sequenced using Illumina HiSeq 4000 or NextSeq 500 platforms (2 × 150 bp). Quality control and low-quality reads were filtered out using Fastp (Chen et al., 2018), and genome-guided alignment was performed to the *Clonorchis sinensis* (GCF_000317415) using Bowtie2 version 2.3.4.1 (Langmead and Salzberg, 2012). Reads that did not map to the host genome were assembled using Trinity version 2.8.5 (Grabherr et al., 2011), and the assembled contigs were analyzed with BLASTn. The virome of the material was also investigated using VirFind (Ho and Tzanetakis, 2014).

## Results

### CYVaV RNA Discovery and Sequence Properties

**Figure 2:**
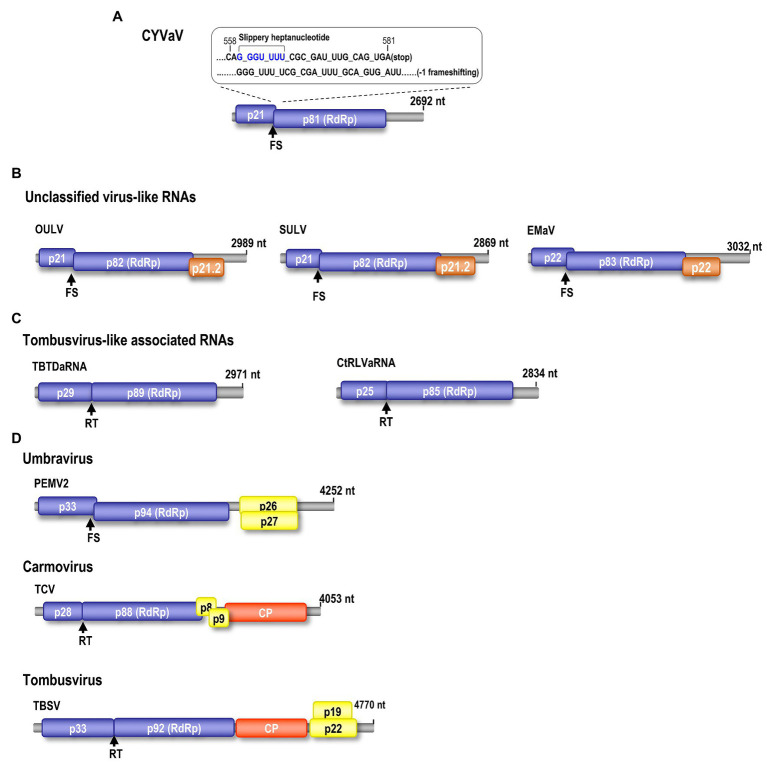
Genomic organization of CYVaV. The CYVaV genome contains two open reading frames (ORFs): ORF1 (replication-required protein, nt 9–581, 21.5 kDa), ORF2 (RNA-dependent RNA polymerase, RdRp, nt 9–2,191, 81 kDa). ORF2 is predicted to be expressed by −1 frame shifting at nucleotide position 561 **(A)**. Comparison of gene organization of selected viruses and virus-associated RNAs in the *Tombusviridae*: Unclassified virus-like RNAs; opuntia umbra-like virus (OULV), sugarcane umbra-like virus (SULV), and Ethiopia maize-associated virus (EMaV; **B**). Tombusvirus-like associated RNAs; tobacco bushy top disease-associated RNA (TBTDaRNA), carrot red leaf virus associated RNA (CtRLVaRNA; **C**). Umbravirus; PEMV2. Carmovirus; turnip crinkle virus (TCV). Tombusvirus; tomato bushy stunt virus (TBSV; **D**). FS, −1 ribosome frameshift recoding. RT, readthrough ribosome recoding.

Phloem rich bark scrapings from stems of a YV-920C graft-inoculated “Etrog” citron seedling expressing typical yellow vein symptoms (Figures 1A–D) were processed for dsRNA isolation and enrichment (Valverde and Nameth, 1990; Kwon et al., 2007). Analysis of dsRNA enriched preparation from the symptomatic plant revealed that symptomatic plant contained a distinct dsRNA of smaller size than 3.0 kb on 1% agarose gel, which not found in healthy plant (Figure 1E). The dsRNA band was gel purified and used for further analysis as described above. The cDNA library constructed from the enriched dsRNA was cloned, and the nucleotide sequences of 48 cDNA clones were assembled using overlapping sequences (Vector NTI Advance 10-InforMax). The identified sequence gaps were filled by RT-PCR, and the 3' and 5' ends were determined by RACE–PCR based on terminal deoxynucleotide transferase poly(G)-tailed cDNA (Kwon et al., 2014). The assembled full-length sequence of the contig comprised of 2,692 nt, which was then deposited in GenBank (JX101610) and provisionally named CYVaV. ORF prediction showed that the contig contains two ORFs flanked by a short 5' untranslated region (UTR) of 8 nt and 3' UTR of 531 nt (Figure 2A). ORF1 extends from 9 to 581 nt and encodes a protein of 190 aa with a predicted molecular weight of 21.5 kDa, whereas ORF2 extends from 9 to 2,191 nt and is predicted to be translated *via* a −1 ribosomal frameshift leading to a fusion protein product of 717 aa (81 kDa). Sequence analysis of the overlap region between ORF1 and ORF2 revealed the presence of a slippery haptanucleotide, GGGUUUU560-566 typically responsible for the −1 frameshifting event (Figure 2A; Brault and Miller, 1992).

BLASTn analysis of ORF 1 did not identify any accessions currently deposited in the GenBank with similar nucleotide sequences. Based on BLASTx analysis, this protein is an apparent ortholog of proteins encoded by unclassified virus-like RNAs such as opuntia umbra-like virus (OULV; 63% aa identity with 95% coverage, *E* value 4e-68), sugarcane umbra-like virus (SULV; 44% aa identity with 89% coverage, *E* value 1e-35), and Ethiopia maize-associated virus (EMaV; 39% aa identity with 95% coverage, *E* value 1e-31).

BLASTn analysis of the nucleotide sequence of ORF2 showed 75% identities (89% coverage, *E* value 0.0) only to the OULV ortholog. Based on BLASTp analysis, CYVaV RdRp shares the highest aa sequence identities to the OULV orthologs 79% (74% coverage, *E* value 0.0) and to the orthologs of EMaV (65% aa identity with 67% coverage, *E* value 0.0) and SULV (60% aa identity with 96% coverage, *E* value 0.0). Lower aa sequence identities (>50%) were shared with orthologs of unclassified virus-like RNAs, such as papaya virus Q (PpVQ; 45% aa identity with 60% coverage, *E* value 4e-108), babaco virus Q (BabVQ; 46% aa identity with 60% coverage, *E* value 2e-122), and papaya meleira virus 2 (PMeV2; 43% aa identity with 60% coverage, *E* value 5e-111). Low aa sequence identities (<40%) were shared with orthologs of umbraviruses, carmoviruses, tombusvirus-like associated RNAs (tlaRNAs), and other members of the family *Tombusviridae* (Supplementary Table 2).

**Figure 3:**
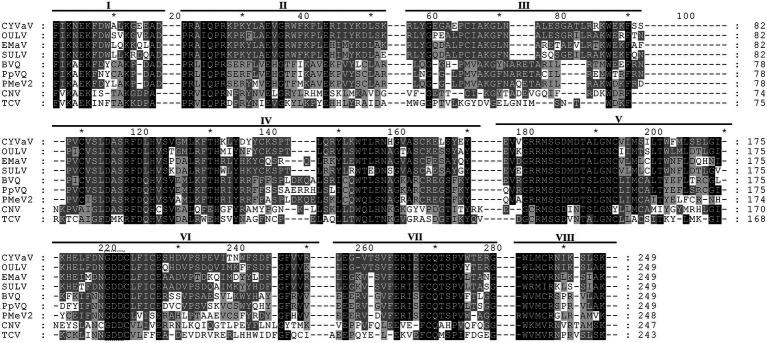
Alignment of the amino acid sequences of the conserved domains of RdRp of CYVaV and related unclassified virus-like RNAs of the family *Tombusviridae*. Opuntia umbra-like virus (OULV), Ethiopia maize-associated virus (EMaV), sugarcane umbra-like virus (SULV), babaco virus Q (BVQ), papaya virus Q (PpVQ), and papaya meleira virus 2 (PMeV2). The viruses cucumber necrosis virus (CNV, *Tombusvirus*) and TCV (*Carmovirus*) were used as guidelines for the identification of the eighth conserved RdRp domains (marked as I–VIII) per Koonin (1991). The numbering above the alignment is arbitrary, starting from the first aligned residue. Amino acids present in at least two sequences were highlighted with different shades of gray as prepared by GeneDoc v2.7.00 (Nicholas and Nicholas, 1997).

Citrus yellow-vein associated virus genome organization is similar to that of unclassified virus-like RNAs and tlaRNAs of the family *Tombusviridae*, with a few exceptions. The virus-like RNAs, OULV, SULV, and EMaV are predicted to contain an additional ORF that overlaps with the end of ORF2 and encodes a protein of unknown function (Figure 2B; Felker et al., 2019). The tlaRNAs, tobacco bushy top disease-associated RNA (TBTDaRNA) and carrot red leaf virus associated RNA (CtRLVaRNA) use ribosome readthrough to express their RdRp domains, similar to tombusviruses and carmoviruses (Figure 2C; Campbell et al., 2020). Finally, CYVaV has no ORFs that correspond to the movement and stabilizing proteins ORFs found in many tombusvirids (Figure 2D). The C' terminus of the fusion protein encodes the RdRp containing all +ssRNA viruses conserved motifs (pfam00998; Figure 3; Kamer and Argos, 1984; Koonin, 1991).

The presence of the CYVaV on the tested YV-920C “Etrog” citron was verified by RT-PCR using two sets of CYVaV sequence specific primers (details specified above) and recovering the expected size and sequence amplicons (data not shown).

### CYVaV 5' and 3' Sequences and Structures

**Figure 4:**
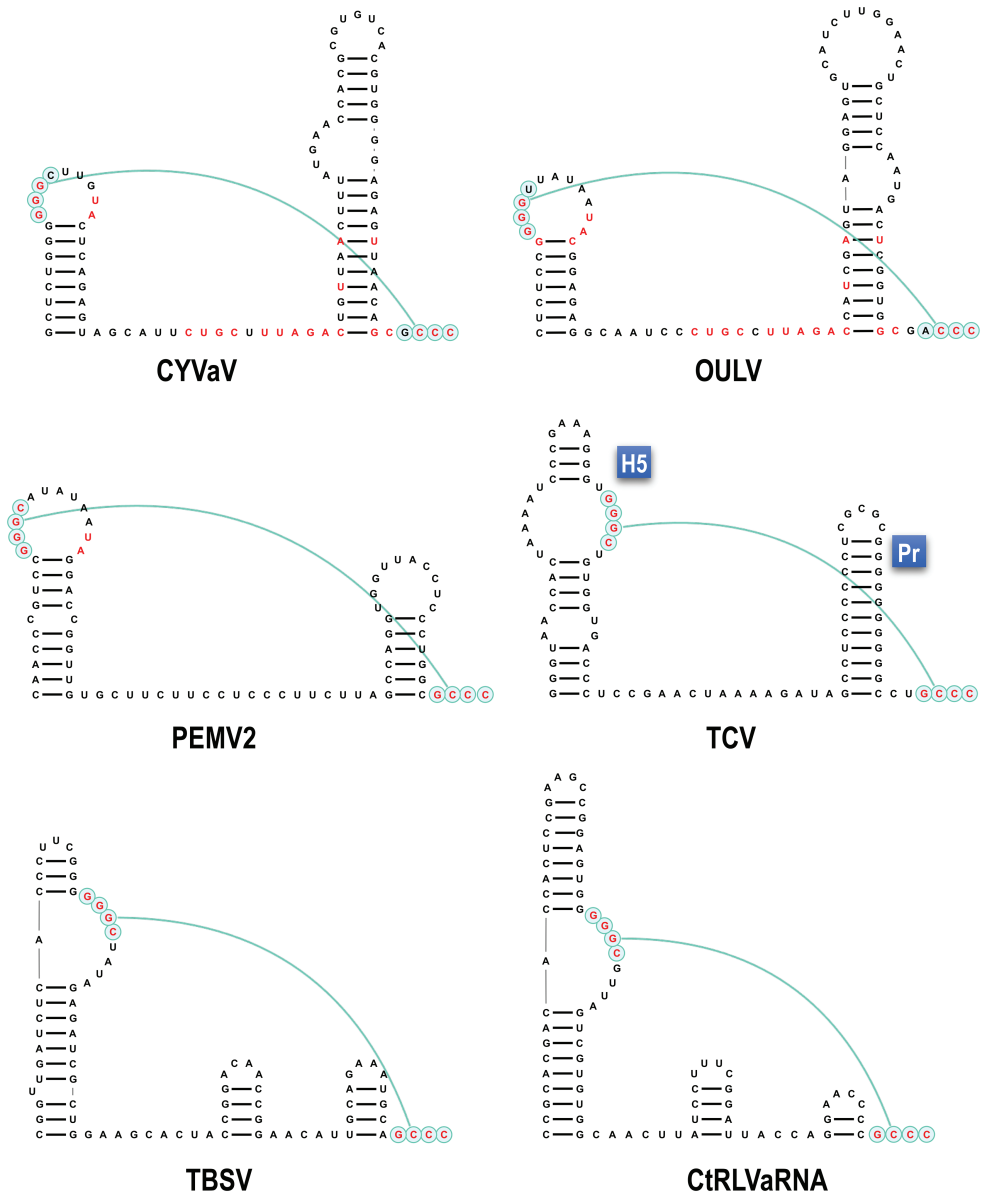
Comparison of 3' terminal features between CYVaV, OULV, and selected viruses in the *Tombusviridae* and tombusvirus-like associated RNAs (tlaRNAs). Known structures in Umbravirus; PEMV2, Carmovirus; TCV, and Tombusvirus; TBSV and predicted structures in CYVaV, OULV, and the tlaRNA, carrot red leaf virus associated RNA (CtRLVaRNA), are shown. Similar sequences are in red. Pseudoknot is in blue. H5, penultimate hairpin; Pr, 3' terminal hairpin.

Analysis of the sequence at the 3' end of the CYVaV genome revealed two hairpins in similar locations as in umbravirus and carmovirus, known as H5 and Pr (Figure 4; Gao et al., 2014; Simon, 2015). The putative CYVaV H5 hairpin most closely resembles umbravirus H5 and contains the conserved 5'GGGC motif in an identical location that pairs with 3' terminal sequences in a pseudoknot known as ψ1 in carmovirus and tombusvirus (Figure 4, in red; Pogany et al., 2003; Mccormack et al., 2008). Two additional adjacent residues in the H5 loop (UA) that are conserved in all umbraviruses are also present in CYVaV (Figure 4, in red). In addition, CYVaV contains the identical umbravirus 3' terminal consensus sequence GCCC-OH 3' (Figure 4, in green). These 3' end features are also present in OULV, but not in tlaRNAs, whose 3' structures resemble those of tombusviruses (Figure 4). The H5 and Pr 3' terminal hairpins are separated by a single-stranded <20 nt linker sequence in CYVaV, umbraviruses, and carmoviruses (Figure 4; Gao et al., 2014).

**Figure 5:**
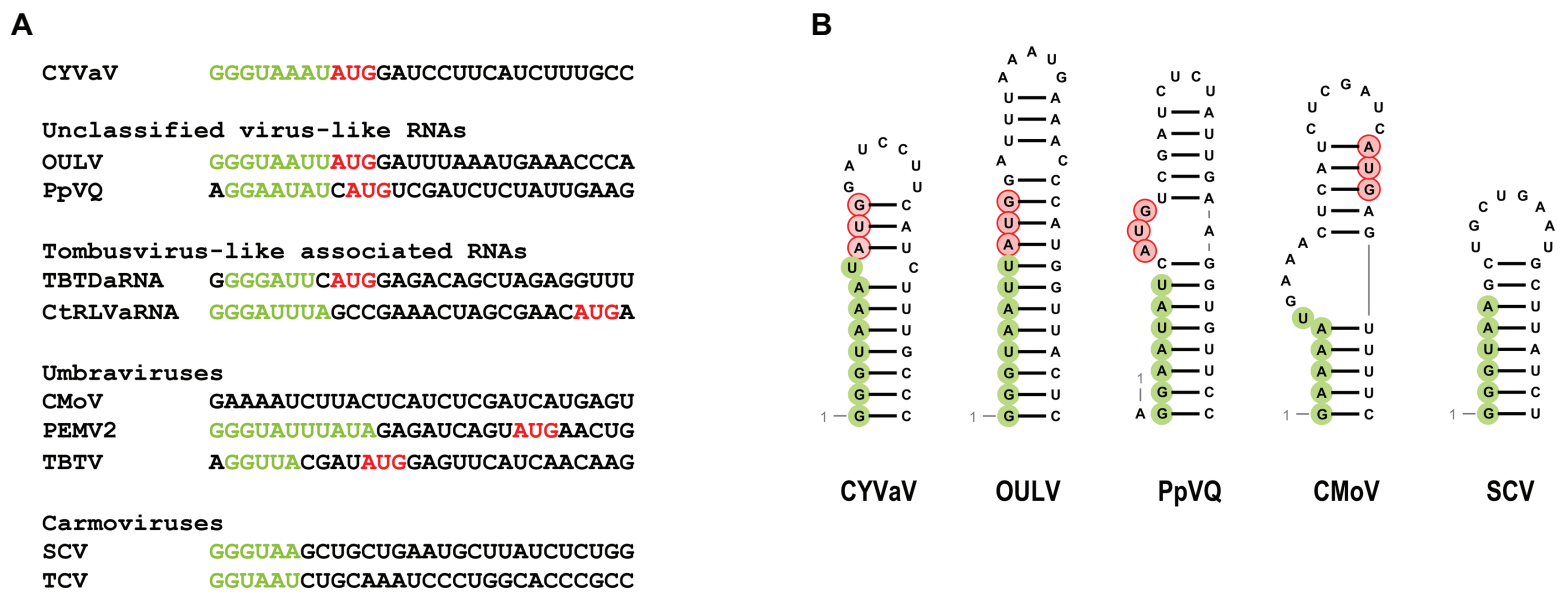
Sequences **(A)** and the predicted secondary structure **(B)** at the 5' ends of CYVaV and selected viruses and virus-associated RNAs. Carmovirus consensus sequence (CCS) is in green and initiation codons for 5' ORFs are in red. Unclassified virus-like RNAs; OULV and PpVQ. Tombusvirus-like associated RNAs; tobacco bushy top disease-associated RNA (TBTDaRNA) and carrot red leaf virus associated RNA (CtRLVaRNA). Umbravirus; carrot mottle virus (CMoV), PEMV2, and tobacco bushy top virus (TBTV), Carmovirus; saguaro cactus virus (SgCV) and turnip crinkle virus (TCV).

Similar to umbraviruses, CYVaV has a short (8 nt) 5' UTR containing a perfect carmovirus consensus sequence (CCS; 5'-GGGUAAAU-3'; Figure 5A, in green; Guan et al., 2000). The CCS is also conserved in OULV, PpVQ, and the tlaRNAs TBTDaRNA and CtRLVaRNA. The CYVaV CCS is predicted to be incorporated into a stem-loop, similar to the stem-loops present in the 5' termini of some umbraviruses and carmoviruses (Figure 5B; Gibbs et al., 1996; Weng and Xiong, 1997).

### CYVaV Phylogenetic Relationships

**Figure 6:**
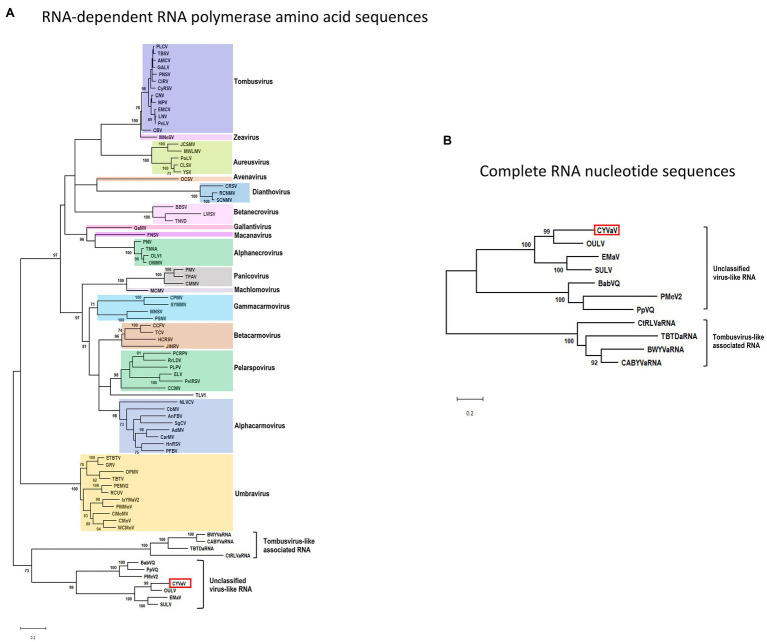
Phylogenetic relationships of CYVaV and members of the family *Tombusviridae*. Maximum likelihood phylogenetic trees of the conserved RdRp amino acid sequences **(A)** and complete RNA nucleotide sequences **(B)** were constructed using MEGA X program (Kumar et al., 2018). Branch lengths represent genetic distances. The numbers on the branches indicate bootstrap percentage based on 1,000 replications. Major virus genera are highlighted in different colors. The full names of the viruses and accession numbers used in the phylogenetic analysis are listed in Supplementary Table 3.

Phylogenetic analyses were performed using the aa sequences of the conserved domains of the RdRps (Koonin, 1991; Figure 6A) of selected members of the family *Tombusviridae* (Supplementary Table 3). CYVaV forms a highly supported cluster with the unclassified virus-like RNAs. This cluster was divided into two clades, one including CYVaV, OULV, SULV, and EMaV and the other with PMeV2, PpVQ, and BabVQ (Figure 6A). tlaRNAs, such as BWYVaRNA, TBTDaRNA, CABYVaRNA, and CtRLVaRNA, which are similar in genome size and organization to CYVaV but differ in their 3' structures and read-through expression mechanism, were grouped in a related clade but in a different cluster from CYVaV (Figure 6A). The pairwise comparison of the aa sequences of the CYVaV RdRp and its orthologs of the related virus-like RNAs, tlaRNAs and viruses showed identities below the 75% demarcation criterion for a new genus within the family *Tombusviridae* (Table 1; Scheets et al., 2015).

**Table 1:** Sequence identities of pairwise comparisons of complete amino acid sequence of RNA-dependent RNA polymerase (RdRp) of citrus yellow-vein associated virus (CYVaV) with phylogenetically related members of the family *Tombusviridae*.

Genus	*Unclassified virus-like RNAs* ^1^						Tombusvirus-like associated RNA^2^		*Umbravirus* ^3^			*Carmovirus* ^4^		*Necrovirus* ^5^	
Species	OULV	SULV	EMaV	PMeV2	BabVQ	PpVQ	TBTDa	CtRLVa	TBTV	CMoV	PEMV2	TCV	SgCV	OLV1^6^	TNV-A^6^
CYVaV RdRp % identity^1^	73.5	59.2	58.3	30.7	34.0	33.3	25.9	23.7	31.8	31.4	28.6	24.7	24.1	24.4	23.7

1 Pairwise comparisons were performed using Megalign (Lasergene, DNAstar, Madison, WI, United States).

2 OULV, opuntia umbra-like virus; SULV, sugarcane umbra-like virus; EMaV, ethiopia maize-associated virus; PMeV2, papaya meleira virus 2; BabVQ, babaco virus Q; and PpVQ, papaya virus Q.

3 TBTDa, tobacco bushy top disease-associated RNA; CtRLVa, carrot red leaf virus associated RNA.

4 TBTV, tobacco bushy top virus; CMoV, carrot mottle virus; and PEMV2, pea enation mosaic virus 2.

5 TCV, turnip crinkle virus; SgCV, saguaro cactus virus.

6 OLV1, olive latent virus 1; TNV-A Tobacco necrosis virus A.

The phylogenetic tree inferred using the full-length nucleotide sequences of CYVaV and related unclassified virus-like RNAs and tlaRNAs was in agreement with the RdRp conserved motifs topology (Figure 6B).

### CYVaV Systemic Infection and Symptom Development Is Independent of Any Known Graft-Transmissible Citrus Virus or Viroid

In the first of the three-serial graft-transmission experiments (2009 experiment #3163), the YV-920C “Etrog” citron plant used in the dsRNA analysis served as the source of inoculum. The two subsequent experiments (2016 and 2020 experiment #3358) were graft-inoculated from a selected plant indicator expressing only yellow symptoms (sequential order of inoculation: YV-920C followed by 2009 experiment #3163‐ tree 02 followed by 2016 experiment #3358‐ tree 70 followed by 2020). In all three experiments, the cumulative total number of yellow vein indicators expressing typical yellow vein symptoms was 6 of 6 for “Etrog” citron and 22 of 22 for Mexican Lime. On the other hand, none of the vein enation indicators, sour orange (0/16) and Mexican lime (0/16) expressed vein enation symptoms. In all experiments, positive controls began expressing symptoms within 7 weeks post graft-inoculation whereas the non-inoculated controls remained asymptomatic for the duration of the experiments.

VirFind based bioinformatic analysis for the identification and discovery of RNA-seq reads related to all potential viruses or viroids, on “Etrog” citron (2009 experiment #3163‐ tree 02) and Mexican lime (2016 experiment #3358‐ tree 70) expressing yellow vein symptoms, identified several contigs of 2,686–2,692 nt length containing the near full-length genome of CYVaV. VirFind analysis did not identify sequences of any known graft-transmissible viral pathogens of citrus (Table 2). Multiple contigs of other sequences such as citrus endogenous pararetrovirus were identified in all tested samples, including the non-inoculated controls, indicating that they, most likely, do not affect symptom expression (Table 2).

**Table 2:** RNA-seq and VirFind identification of graft-transmissible virus and viroids of citrus associated with citrus plants expressing yellow vein symptoms.

Sample^1^	Citrus Host^2^	Treatment^3^	Symptoms (severity, 1–5)^4^	Reads before filtering (millions)	Reads after filtering (millions)^5^	Citrus viral pathogen identified^6^	Pathogen genome size (nt)^7^	Contigs (length, nt)
**Yellow vein**								
#3163-02	Ctrn	YV-920C	yellow vein (+5)	90.99	73.87	CYVaV	2,692	1 (2,682)
#3358-70	ML	#3163–02 YV920C	yellow vein (+2)	103.57	80.13	CYVaV	2,692	3 (2,394, 2,685, and 2,686)
**Controls**								
VI 357–1,005,966	Ctrn	NI	NS	18.07	17.24	None	N/A^8^	0
VI 419–1,005,760	ML	NI	NS	16.16	15.37	None	N/A	0
VI 222–1,005,793	SwO	NI	NS	26.5	25.43	None	N/A	0
#3253–06	SwO	VE709	vein enation (+4)	29.31	28.29	CVEV	5,983	1 (5,929)
#3347–07	SwO	P202	young leaf pattern (+1)	35.84	33.26	CPsV	RNA1: 8,186 RNA2: 1,645 RNA3: 1,447	RNA1: 12 (213-1,099) RNA2: 1 (1,617) RNA3: 1 (1,422)
#3323-337-IPPN 786	SwO	Mix	NBT	31.3	30.97	CTV CEVd HSVd CDVd	19,296 372 302 294	78 (34-8,502) 1 (372) 2 (271) 2 (294)^9^

1 Citrus clonal protection program (CCPP) experiment number (#), CCPP variety indexing number (VI)-registration number, and CCPP introductory plant propagation number (IPPN).

2 Ctrn: “Etrog” citron (*Citrus medica* L.); ML, mexican lime [*Citrus aurantifolia* (Christm.) Swingle]; SwO, sweet orange [*C. sinensis* (L.) Osbeck].

3 YV920C, yellow vein disease accession, CCPP Disease Bank (DB); NI, non-inoculated; VE709: vein enation disease accession, CCPP-DB; P202, psorosis disease accession, CCPP-DB; and Mix, mixture of graft-transmissible pathogens of citrus.

4 Severity, +1 mild – +5 severe; NBT, No biological test. Sample was collected from original budwood for laboratory tests only because of high risk out of state citrus variety introduction (see also note 8).

5 Low-quality reads were filtered out with Fastp (Chen et al., 2018).

6 CYVaV, citrus yellow-vein associated virus; CVEV, citrus vein enation virus; CPsV, citrus psorosis virus; CTV, citrus tristeza virus; CEVd, citrus exocortis viroid; HSVd, Hop stunt viroid; and CDVd, Citrus dwarfing viroid.

7 Genome sizes are reported from GenBank accessions. CYVaV: JX101610; CVEV: NC_021564.1; CPsV: NC_006314.1-NC_006314.3; CTV: NC_001661.1; CEVd: NC_001464.1; HSVd: NC_001351.1; and CDVd: NC_003264.1.

8 N/A, not applicable.

9 Sample #3323-337-IPPN 786 also contained contigs of Candidatus Liberibacter asiaticus (16 contigs, 204–1,289 nt).

## Discussion

To determine the nature of the CYDV pathogen, we performed multiple screenings of citrus trees with characteristic symptoms. This demonstrated that a novel RNA molecule, termed CYVaV, is associated with CYVD symptom development independently of any known graft-transmissible viral pathogens of citrus.

Citrus yellow-vein associated virus is related but appears to be distinct among the members of the family *Tombusviridae*. For example, the CYVaV RNA genome of 2,692 nt is smaller than the umbra-, carmo-, and tombus‐ viruses (genomes >4,000 nt) as well as the tlaRNAs (genomes >2,800 nt). In addition, while CYVaV contains two ORFs, like some tombusvirids, encoding the required replication components, it is missing the overlapping movement protein ORFs found in all umbraviruses (Figure 2) and the predicted ORF overlapping ORF2 of the unclassified virus-like RNAs (Ryabov et al., 1999, 2001; Taliansky et al., 2003; Taliansky and Robinson, 2003; Felker et al., 2019).

Despite its distinct genome size and organization CYVaV shares important RNA structural features on its 3' and 5' ends with members of the family *Tombusviridae* (Figures 4, 5). CYVaV, umbraviruses, carmoviruses, and some other tombusvirids, contain two conserved hairpins at their 3' ends (Figure 4; Gao et al., 2014; Simon, 2015). The penultimate hairpin, known as H5, contains the conserved motif (5'GGGC/U) in its terminal loop (CYVaV and umbraviruses) or in an internal symmetrical loop (carmoviruses) or an internal asymmetrical loop (tombusviruses), which pairs with 3' terminal sequences in a pseudoknot known as ψ_1_, as demonstrated in carmovirus turnip crinkle virus (TCV; Mccormack et al., 2008) and tombusvirus tomato bushy stunt virus (TBSV; Pogany et al., 2003). In CYVaV, umbraviruses, and carmoviruses, the conserved 3' terminal sequences are 5'GCG/ACCC-OH and 5'CCNGCCC-OH, respectively (ψ_1_ pairing partner is underlined).

Citrus yellow-vein associated virus, umbraviruses, and carmoviruses have short 5' UTRs. The 5' ends of most umbravirus and carmovirus genomic and subgenomic RNAs begin with a conserved sequence known as the carmovirus consensus sequence (CCS); G_1-3_A/U_3-8_. This sequence usually begins with three guanylates as in the case of CYVaV (Figure 5A, in green; Guan et al., 2000). The CYVaV RACE analyses and structure similarities to the 5' and 3' ends of tombusvirids strongly suggests that approximately 2.7 kb RNA sequence is the full-length sequence of this infectious agent.

Recent studies on various plant hosts, reported small viruses and virus-associated RNAs of approximately 2.8–4.5 kb in size containing two or more ORFs (Mo et al., 2011; Quito-Avila et al., 2015; Sa Antunes et al., 2016; Felker et al., 2019; Campbell et al., 2020; Yoshida, 2020; Tahir et al., 2021). CYVaV has been referenced by many of these reports to be related to such small virus-like RNAs (Quito-Avila et al., 2015; Sa Antunes et al., 2016; Tahir et al., 2021). However, CYVaV remains distinct among them because of the smaller size (approximately 2.7 kb) and organization of its genome and putative expression mechanism of its two ORFs (−1 ribosomal frameshift; Figure 2) as well as the low aa sequence similarity of its ORF1 encoded protein and RdRp (i.e., <74%).

The combination of structural elements at the virus ends and the phylogenetic analysis of tombusvirids and tombusvirid-like RNAs provide an insight into the evolution of the tombusvirids at-large. The structures of all the viruses are highly conserved and presumed present in the last common ancestor of the group. In addition, it is well-established that ORF1 in all viruses in the family *Tombusviridae* codes for a replication required protein that is involved in membrane reorganization and interaction with host factors (Rajendran and Nagy, 2003; Pogany et al., 2005; Kovalev et al., 2016). The fusion ORF1/2 in both groups are expressed *via* a −1 ribosomal frameshift. The RdRp phylogram clearly shows that the CYVaV-like viruses are ancestral to the group. They, along with tlaRNAs, do not have signature virus genes other than the RdRp. One hypothesis is that a member from the group acquired movement-associated genes from a host or another virus that transformed it to a bone fide virus: possibly an ancestral umbra-like virus. Co-infection with another virus, presumably a luteo-like (Adams et al., 2011) provided the umbra-like virus with a coat protein forming an ancestral virus to the modern tombusvirids. Luteovirid coat proteins are ancestral to their tombusvirids counterpart (Adams et al., 2011) and both have a picorna-like jelly roll folding (Byrne et al., 2019). We therefore have a plausible hypothesis of how genetic elements similar to CYVaV evolve to form bone fide plant-infecting +sense RNA viruses.

Citrus yellow-vein associated virus is closely related to unclassified virus-like RNAs in the family *Tombusviridae*. Among these unclassified virus-like RNAs, CYVaV was grouped as most related to OULV. tlaRNAs, such as BWYVaRNA, TBTDaRNA, CABYVaRNA, and CtRLVaRNA, which are similar in genome size and organization to CYVaV were classified in a different cluster from CYVaV. Campbell et al. (2020) reported that tlaRNAs clustered into three groups which formed a monophyletic clade with members of the *Tombusviridae*. Topologies of trees generated in the current study support the hypothesis that the unclassified virus-like RNAs, including CYVaV, and the tlaRNAs are representing ancestral RNAs within the family *Tombusviridae*.

The series of bio-indexing experiments and the RNA-seq analysis indicated that CYVaV is capable of inducing systemic yellow vein symptoms in the absence of CVEV or any other known citrus virus or viroid. These results are in agreement with the early reports for one of the four original CYVD limequat (citrus × floridana) field trees, that appeared to be free of either psorosis or exocortis and it was not contaminated with other known viruses (Weathers, 1960, 1961). However, we cannot exclude the possibility that at some point in time, CYVaV was associated with another citrus virus, such as CVEV, that could have acted as a helper virus for CYVaV encapsidation and subsequent aphid transmission (Da Graça and Maharaj, 1991; Clark and da Graça, 2000) that resulted to the original introduction of CYVaV into citrus. It is also possible that after the original vector transmission event the helper virus was lost naturally, for instance because of high temperatures. A series of thermotherapy experiments clearly demonstrated that the CYVD pathogen is heat tolerant whereas other citrus pathogens are not. More specifically, the CYVD pathogen was not eliminated from citrus plants after 12 weeks of exposure to 40°C (16 h/day) followed by 30°C (8 h/night) while under the same conditions most citrus pathogens, including the one causing vein enation, were eliminated from citrus as soon as 8 weeks (Calavan et al., 1972). In addition, it is possible that during one of the multiple graft-inoculations that took place in the CCPP greenhouse since 1969 for the *in planta* maintenance of the YV-920 isolate, only the CYVaV was passed from the source plant to the progeny (e.g., inoculum survival, uneven distribution of pathogens etc.) while any helper virus was not moved forward to the plants of the YV-920C isolate used in our experiments. This could also explain the positive and negative biological indexing results for vein enation recorded previously for the yellow-vein isolates at the CCPP (data not shown). Further studies are required to prove such hypotheses and more importantly to characterize sequence and structural features that may support, the conjectural CYVaV encapsidation and insect transmission but also to exclude the possibility that one or more unknown virus(es) are supporting the CYVaV biology and the phenotypes observed in this study (Kwon et al., 2005).

The biological observations and the RNA-seq data in combination with the genome organization and molecular characterization presented here, indicate that CYVaV has potentially unique properties among the members of the family *Tombusviridae*. On that basis, additional research efforts are currently focused on fulfilling Koch’s postulates using full-length infectious clones, studying the replication and systemic movement of CYVaV in plants, setting up field trials to evaluate CYVaV effects on tree growth and yield in single and mix infections with other citrus pathogens, developing CYVaV as a virus-induced gene silencing (VIGS) expression system, a potential RNA interference (RNAi) based approach for citrus pest and disease management (Simon et al., 2020).

## Data Availability Statement

The datasets presented in this study can be found in online repositories. The names of the repository/repositories and accession number(s) can be found in the article/Supplementary Material.

## Author Contributions

S-JK, IT, AS, and GV conceived and designed the experiments. S-JK discovered and S-JK, SB, KG, TH, FO, and MR characterized the CYVaV RNA. AS performed structural sequence analysis. SB, KG, and GV conducted bio-indexing experiments and TD performed RNA-seq experiments. S-JK and GV wrote the first draft of the manuscript and all authors contributed to manuscript revision, read, and approved the submitted version.

### Conflict of Interest

The authors declare that the research was conducted in the absence of any commercial or financial relationships that could be construed as a potential conflict of interest.
