# Supplementary material for: Complete Nucleotide Sequence, Genome Organization, and Comparative Genomic Analyses of Citrus Yellow-Vein Associated Virus (CYVaV)
Source: Front Microbiol. 2021 Jun 8;12:683130. doi: 10.3389/fmicb.2021.683130 (PMC8218546; doi:10.3389/fmicb.2021.683130)
Supplement: Supplementary file 1 [file Table_1.DOCX]

**Supplemental Table 1.** Information on assembled contigs and primer locations used for filling the sequence gap between contigs

| No | Sequence | Size |
| --- | --- | --- |
| Contig #1 | Catcatgccagacaggtgtttcgagcatcaactagcttctcaagagaggtggttcgcgctgctcgtagatgggttaccatgcccaccagtcgccatgcatatgacttttcaacgagtctaggcattgtgattgctgagcctgcagctcgtttacgacgccgtctgccctctgtacgaaagtgcgcagagaagttagtagtccacaagcaagtcgacactttggtggacgaatggtgctctggaattcccaaccctgatatcgtagaagttggttgggcactccgtctgagggaccgtttcggtcttcctcccgcttctgagcctacccggctcagtggtgagagatgggtgctcaaacaactcaatggggtagatcctgagtcatggaatgctgatcttggtaggtcagttcatatccaaggagactacgccccagggaggaatgcccatatcgctcaggtcgcggcgaccttgtggttaactaggaccttgcatgacaaggccttggctcgccaccagggttttcgcgatttgcagtgattggggtcgacgggctagaggcaaaagcagtgcctctagcttctggactccgactgcttccggttccgcgacccggacaaagtcgacgactgtctcagaccttgttacttccaacacctcgtgctcaattcgtgaatcacgcgtgctcggctaacaaccttggacgtgtgatgaccacacgtgtgttgcagtacaagggccgagatccgatccttccctcttctgaagcccttcaccgacttaaccttcggatagctgagctatataggtcta***gaccttctaccgtctatccatta*^*^**agttatgaagggtttctcaattgctatgaaggccgacagcgtactcgttacgcccaagccgtcgagcagttgatgcggtccactcttgagccga | 930 nt |
| Contig #2 | Ttgactgggcgttgaaaggggaggaggctgatcctcgagcaatccaaccaaggaagccgaaatatttggctgaggttggacggtggttcaaacctttggagcgaa***tcatctacaaggatctcagta*^#^**aaaggttgtatggtgagggtgctgagccgtgtatcgccaaaggcctaaatgcattagaatctggagcgactttgaggcgcaaatgggagaagttttcttctccagtttgcgtttctctcgacgcttccaggttcgacctgcatgtaagcgttggcatgctaaagttcacacacaagctatatgactattactgtaagtctcccactctccagcgctatctcaaatggacactccgcaaccatggcgtcgcctcctgcaaagaattgtcatatgagtatgaggttgttggccggagaatgagtggtgacatggacactgcattgggcaactgcgtcattatgtcgatacttacatggtttatgcttagtgaacttggcattaagcatgaattattcgataatggtgacgattgtttgttcatttgcgagtctcacgacgtccccagccccgaggtaattacaaactggttttcggactttgggtttgtggttaggttggaaggcgtcacgtccgtgtttgagcgtattgagttttgccaaacttccccagtatggactgagaggggttggctgatgtgtaggaatattaagtcattgagtaaagaccttacgaatgttaattcgtgcacgggctccacgattgaatatacccactggttgaaagcagtgggaaagtgcgggtcaatactcaatgctggtgtacctatatttcagtcctttcacaacatgctggaaaggcttggcactaactctcgtattgatcgaggggttttcttcaaatcagggctagttaatctcattcgtgggatggacaggcagcctgacgttgacatcactacttccgctcggctttctttcgaagtggcattcgggataacacccgggatgcaattggctattgaacggtactatgactctgtcatgggctcgctgagtaaaatagaaacaactaagtggccaattgaactaagaaaggaatacgaacacggaagtgagtggtacgaggacttaggcgtcctaggatgaatagggtcattggtttaccgatgatacctgttcagaataggattgctcgagcttcgttggttagggtaactcacataccttcttccataactggaaaaggtcgtgtgagcaacctaaccagttaatgtaggtgtctttccgtatctagtcacgatggtaagcaacccgtttatctgtacggcgctcacccgtgggtaggaaggtgaaggttttgtgtcctttaggtcttggacagtctgcgggcttgggaacgacgccccgctagcaacgtactgctctcctaccggactggtagcttaattgtcatcttggagcgatagcactgtgggcctcacccttcgcgcgttggacgtgttgcgtgccccccacagatttgtgaaactctatggagcagttccgcgagccagaagggaggatggccgcctggcgtaatccaggagctctggggggcttgtactcagagtagcattctgctttagactgttaactttatgaaccacgcgtgtcacgtggggagagtta | 1673nt |

*****, Forward primer location for filling the gap sequence

**^#^**, Reverse primer location for filling the gap sequences

**Supplemental Table 2.** Citrus yellow-vein associated virus RNA-dependent RNA polymerase amino acid sequence identities to related viruses and virus-like RNAs of the family *Tombusviridae*. Alignment and comparison performed by BLASTp*.*

| **Virus name** | **Acronym** | **Max**  **Score** | **Total**  **Score** | **Percent**  **Identity** | **Query**  **Cover** | **E value** | **Accession**  **Length** | **Genus** |
| --- | --- | --- | --- | --- | --- | --- | --- | --- |
| Opuntia umbra-like virus | OULV | 884 | 884 | 78.8% | 74% | 0 | 540 | Unclassified |
| Ethiopia maize-associated virus | EMaV | 659 | 659 | 65.3% | 67% | 0 | 489 | Unclassified |
| Sugarcane umbra-like virus | SULV | 836 | 836 | 60.0% | 96% | 0 | 708 | Unclassified |
| Papaya virus Q | PpVQ | 347 | 347 | 45.0% | 60% | 3.00E-114 | 475 | Unclassified |
| Babaco virus Q | BabVQ | 369 | 369 | 45.8% | 60% | 2.00E-122 | 501 | Unclassified |
| Papaya meleira virus 2 | PMeV2 | 338 | 338 | 42.7% | 60% | 5.00E-111 | 473 | Unclassified |
| Carrot mottle virus | CMoV | 354 | 354 | 39.7% | 73% | 8.00E-113 | 870 | Umbravirus |
| Carrot mottle mimic virus | CMoMV | 379 | 379 | 39.4% | 80% | 9.00E-122 | 882 | Umbravirus |
| Ixeridium yellow mottle associated virus 2 | IxYMaV2 | 372 | 372 | 38.5% | 84% | 3.00E-119 | 872 | Unclassified |
| Groundnut rosette virus | GRV | 350 | 350 | 38.4% | 72% | 3.00E-111 | 852 | Umbravirus |
| Carnation mottle virus | CarMV | 243 | 243 | 38.3% | 54% | 4.00E-72 | 763 | Alphacarmovirus |
| Wild carrot mottle virus | WCMoV | 361 | 361 | 38.1% | 80% | 3.00E-115 | 874 | Unclassified |
| Red clover umbravirus | RCUV | 337 | 337 | 37.9% | 72% | 3.00E-106 | 870 | Unclassified |
| Pea enation mosaic virus 2 | PEMV2 | 320 | 320 | 37.8% | 72% | 5.00E-100 | 845 | Umbravirus |
| Melon necrotic spot virus | MNSV | 273 | 273 | 37.8% | 65% | 6.00E-83 | 791 | Gammacarmovirus |
| Galinsoga mosaic virus | GaMV | 298 | 298 | 37.8% | 66% | 9.00E-93 | 729 | Gallantivirus |
| Opium poppy mosaic virus | OPMV | 342 | 342 | 37.7% | 72% | 3.00E-108 | 865 | Umbravirus |
| Tobacco bushy top virus | TBTV | 358 | 358 | 37.7% | 80% | 3.00E-114 | 865 | Umbravirus |
| Tobacco bushy top disease-associated RNA | TBTDaRNA | 250 | 250 | 37.6% | 61% | 1.00E-74 | 788 | Tombusvirus like associated RNA |
| Saguaro cactus virus | SgCV | 250 | 250 | 37.2% | 61% | 8.00E-75 | 755 | Alphacarmovirus |
| Patrinia mild mottle virus | PMMoV | 347 | 347 | 37.2% | 82% | 9.00E-110 | 877 | Unclassified |
| Thin paspalum asymptomatic virus | TPAV | 268 | 268 | 37.1% | 62% | 3.00E-80 | 947 | Panicovirus |
| Tobacco necrosis virus A | TNV-A | 277 | 277 | 37.1% | 62% | 6.00E-85 | 724 | Alphanecrovirus |
| Ethiopian tobacco bushy top virus | ETBTV | 348 | 348 | 37.0% | 77% | 2.00E-110 | 845 | Umbravirus |
| Cucurbit aphid borne yellows virus associated RNA | CABYVaRNA | 256 | 256 | 36.9% | 66% | 6.00E-77 | 755 | Tombusvirus like associated RNA |
| Japanese iris necrotic ring virus | JINRV | 255 | 255 | 36.7% | 62% | 9.00E-77 | 762 | Betacarmovirus |
| Olive mild mosaic virus | OMMV | 278 | 278 | 36.7% | 63% | 2.00E-85 | 724 | Alphanecrovirus |
| Adonis mosaic virus | AdMV | 248 | 248 | 36.6% | 61% | 3.00E-74 | 767 | Unclassified |
| Pelargonium chlorotic ring pattern virus | PCRPV | 257 | 257 | 36.4% | 62% | 2.00E-77 | 763 | Pelarspovirus |
| Leek white stripe virus | LWSV | 236 | 236 | 36.3% | 54% | 4.00E-70 | 727 | Betanecrovirus |
| Maize white line mosaic virus | MWLMV | 215 | 215 | 36.2% | 53% | 5.00E-62 | 797 | Aureusvirus |
| Carrot red leaf virus associated RNA | CtRLVaRNA | 246 | 246 | 35.8% | 70% | 6.00E-74 | 677 | Tombusvirus like associated RNA |
| Beet western yellows ST9 associated RNA | BWYVaRNA | 269 | 269 | 35.5% | 66% | 3.00E-82 | 726 | Tombusvirus like associated RNA |
| Panicum mosaic virus | PMV | 254 | 254 | 35.5% | 62% | 9.00E-75 | 992 | Panicovirus |
| Cucumber Bulgarian virus | CBV | 207 | 207 | 35.3% | 54% | 3.00E-59 | 790 | Tombusvirus |
| Angelonia flower break virus | AnFBV | 250 | 250 | 35.1% | 65% | 9.00E-75 | 760 | Alphacarmovirus |
| Elderberry latent virus | ELV | 256 | 256 | 35.0% | 63% | 5.00E-77 | 762 | Pelarspovirus |
| Pea stem necrosis virus | PSNV | 250 | 250 | 35.0% | 62% | 5.00E-75 | 750 | Gammacarmovirus |
| Furcraea necrotic streak virus | FNSV | 270 | 270 | 34.9% | 67% | 4.00E-82 | 762 | Macanavirus |
| Trailing lespedeza virus 1 | TLV 1 | 234 | 234 | 34.9% | 53% | 8.00E-69 | 770 | Unclassified |
| Cocksfoot mild mosaic virus | CMMV | 247 | 247 | 34.8% | 61% | 1.00E-72 | 934 | Panicovirus |
| Cardamine chlorotic fleck virus | CCFV | 267 | 267 | 34.8% | 66% | 7.00E-81 | 773 | Betacarmovirus |
| Cucumber leaf spot virus | CLSV | 221 | 221 | 34.7% | 61% | 2.00E-64 | 738 | Aureusvirus |
| Maize chlorotic mottle virus | MCMV | 251 | 251 | 34.7% | 62% | 4.00E-74 | 965 | Machlomovirus |
| Yam spherical virus | YSV | 218 | 218 | 34.7% | 61% | 3.00E-63 | 738 | Aureusvirus |
| Calibrachoa mottle virus | CbMV | 246 | 246 | 34.5% | 72% | 3.00E-73 | 762 | Alphacarmovirus |
| Nootka lupine vein-clearing virus | NLVCV | 221 | 221 | 34.4% | 57% | 2.00E-64 | 774 | Alphacarmovirus |
| Pothos latent virus | PoLV | 226 | 226 | 34.2% | 62% | 2.00E-66 | 740 | Aureusvirus |
| Soybean yellow mottle mosaic virus | SYMMV | 256 | 256 | 34.1% | 66% | 4.00E-77 | 746 | Gammacarmovirus |
| Eggplant mottled crinkle virus | EMCV | 224 | 224 | 34.1% | 62% | 5.00E-65 | 818 | Tombusvirus |
| Lisianthus necrosis virus | LNV | 222 | 222 | 34.1% | 62% | 2.00E-64 | 817 | Tombusvirus |
| Pelargonium line pattern virus | PLPV | 245 | 245 | 34.1% | 65% | 7.00E-73 | 765 | Pelarspovirus |
| Johnsongrass chlorotic stripe mosaic virus | JCSMV | 228 | 228 | 34.0% | 64% | 9.00E-67 | 795 | Aureusvirus |
| Clematis chlorotic mottle virus | CCMV | 238 | 238 | 33.9% | 61% | 2.00E-70 | 756 | Pelarspovirus |
| Grapevine Algerian latent virus | GALV | 224 | 224 | 33.7% | 62% | 6.00E-65 | 818 | Tombusvirus |
| Pear latent virus | PeLV | 220 | 220 | 33.7% | 62% | 1.00E-63 | 818 | Tombusvirus |
| Beet black scorch virus | BBSV | 239 | 239 | 33.6% | 61% | 4.00E-71 | 724 | Betanecrovirus |
| Tobacco necrosis virus D | TNV-D | 239 | 239 | 33.6% | 61% | 6.00E-71 | 725 | Betanecrovirus |
| Hibiscus chlorotic ringspot virus | HCRSV | 255 | 255 | 33.5% | 65% | 9.00E-77 | 735 | Betacarmovirus |
| Rosa rugosa leaf distortion virus | RrLDV | 239 | 239 | 33.5% | 63% | 7.00E-71 | 761 | Pelarspovirus |
| Turnip crinkle virus | TCV | 261 | 261 | 33.4% | 66% | 8.00E-79 | 775 | Betacarmovirus |
| Cucumber necrosis virus | CNV | 224 | 224 | 33.3% | 62% | 6.00E-65 | 818 | Tombusvirus |
| Honeysuckle ringspot virus | HnRSV | 238 | 238 | 33.3% | 64% | 7.00E-70 | 880 | Alphacarmovirus |
| Moroccan pepper virus | MPV | 219 | 219 | 33.3% | 62% | 3.00E-63 | 817 | Tombusvirus |
| Tomato bushy stunt virus | TBSV | 218 | 218 | 33.1% | 62% | 7.00E-63 | 818 | Tombusvirus |
| Artichoke mottled crinkle virus | AMCV | 218 | 218 | 33.1% | 62% | 8.00E-63 | 818 | Tombusvirus |
| Pelargonium ringspot virus | PelRSV | 253 | 253 | 33.0% | 62% | 6.00E-76 | 762 | Pelarspovirus |
| Pelargonium leaf curl virus | PLCV | 219 | 219 | 33.0% | 62% | 4.00E-63 | 852 | Tombusvirus |
| Cowpea mottle virus | CPMV | 242 | 242 | 32.9% | 65% | 4.00E-72 | 717 | Gammacarmovirus |
| Carnation Italian ringspot virus | CIRV | 219 | 219 | 32.8% | 62% | 3.00E-63 | 818 | Tombusvirus |
| Melon necrotic streak virus | MNeSV | 210 | 210 | 32.8% | 62% | 3.00E-60 | 788 | Zeavirus |
| Olive latent virus 1 | OLV1 | 281 | 281 | 32.7% | 80% | 2.00E-86 | 723 | Alphanecrovirus |
| Cymbidium ringspot virus | CyRSV | 219 | 219 | 32.5% | 62% | 2.00E-63 | 818 | Tombusvirus |
| Pelargonium necrotic spot virus | PNSV | 214 | 214 | 32.0% | 65% | 3.00E-61 | 852 | Tombusvirus |
| Pelargonium flower break virus | PFBV | 226 | 226 | 31.9% | 64% | 4.00E-66 | 756 | Alphacarmovirus |
| Oat chlorotic stunt virus | OCSV | 214 | 214 | 31.5% | 66% | 7.00E-62 | 752 | Avenavirus |
| Potato necrosis virus | PNV | 271 | 271 | 31.3% | 81% | 4.00E-83 | 724 | Alphanecrovirus |
| Sweet clover necrotic mosaic virus | SCNMV | 192 | 192 | 30.2% | 66% | 3.00E-54 | 767 | Dianthovirus |
| Carnation ringspot virus | CRSV | 195 | 195 | 29.7% | 66% | 4.00E-55 | 769 | Dianthovirus |
| Red clover necrotic mosaic virus | RCNMV | 205 | 205 | 29.4% | 62% | 1.00E-58 | 767 | Dianthovirus |

**Supplemental Table 3.** List in alphabetical order of viruses and virus-like RNAs in the family *Tombusviridae* used for phylogenetic analysis in this study

| **Virus name** | **Acronym** | **GenBank No.** | **Genus** |
| --- | --- | --- | --- |
| Adonis mosaic virus | AdMV | LC171345 | Unclassified |
| Angelonia flower break virus | AnFBV | DQ219415 | Alphacarmovirus |
| Artichoke mottled crinkle virus | AMCV | X62493 | Tombusvirus |
| Babaco virus Q | BabVQ | MN648673 | Unclassified |
| Beet black scorch virus | BBSV | AF452884 | Betanecrovirus |
| Beet western yellows ST9 associated RNA | BWYVaRNA | L04281 | Tombusvirus like associated RNA |
| Calibrachoa mottle virus | CbMV | GQ244431 | Alphacarmovirus |
| Cardamine chlorotic fleck virus | CCFV | L16015 | Betacarmovirus |
| Carnation Italian ringspot virus | CIRV | KP888563 | Tombusvirus |
| Carnation mottle virus | CarMV | X02986 | Alphacarmovirus |
| Carnation ringspot virus | CRSV | L18870 | Dianthovirus |
| Carrot mottle virus | CMoV | FJ188473 | Umbravirus |
| Carrot mottle mimic virus | CMoMV | U57305 | Umbravirus |
| Carrot red leaf virus associated RNA | CtRLVaRNA | LT608332 | Tombusvirus like associated RNA |
| Clematis chlorotic mottle virus | CCMV | MG660825 | Pelarspovirus |
| Citrus yellow vein associated virus | CYVaV | JX101610 | Unclassified |
| Cocksfoot mild mosaic virus | CMMV | NC011108 | Panicovirus |
| Cowpea mottle virus | CPMV | U20976 | Gammacarmoviruis |
| Cucumber Bulgarian virus | CBV | KJ572966 | Tombusvirus |
| Cucumber leaf spot virus | CLSV | NC007816 | Aureusvirus |
| Cucumber necrosis virus | CNV | M25270 | Tombusvirus |
| Cucurbit aphid borne yellows virus associated RNA | CABYVaRNA | KM486094 | Tombusvirus like associated RNA |
| Cymbidium ringspot virus | CyRSV | X15511 | Tombusvirus |
| Eggplant mottled crinkle virus | EMCV | JQ864181 | Tombusvirus |
| Elderberry latent virus | ELV | NC026239 | Pelarspovirus |
| Ethiopia maize-associated virus | EMaV | MN715238 | Unclassifiec |
| Ethiopian tobacco bushy top virus | ETBTV | KJ918748 | Umbravirus |
| Furcraea necrotic streak virus | FNSV | NC020469 | Macanavirus |
| Galinsoga mosaic virus | GaMV | Y13463 | Gallantivirus |
| Grapevine Algerian latent virus | GALV | AY830918 | Tombusvirus |
| Groundnut rosette virus | GRV | MG646922 | Umbravirus |
| Hibiscus chlorotic ringspot virus | HCRSV | X86448 | Betacarmovirus |
| Honeysuckle ringspot virus | HnRSV | HQ677625 | Alphacarmovirus |
| Ixeridium yellow mottle associated virus 2 | IxYMaV2 | KT946712 | Unclassified |
| Japanese iris necrotic ring virus | JINRV | D86123 | Betacarmovirus |
| Johnsongrass chlorotic stripe mosaic virus | JCSMV | NC005287 | Aureusvirus |
| Leek white stripe virus | LWSV | X96560 | Betanecrovirus |
| Lisianthus necrosis virus | LNV | DQ011234 | Tombusvirus |
| Maize chlorotic mottle virus | MCMV | X14736 | Machlomovirus |
| Maize white line mosaic virus | MWLMV | NC009533 | Aureusvirus |
| Melon necrotic spot virus | MNSV | M29671 | Gammacarmovirus |
| Melon necrotic streak virus | MNeSV | NC007729 | Zeavirus |
| Moroccan pepper virus | MPV | JX197071 | Tombusvirus |
| Nootka lupine vein-clearing virus | NLVCV | EF207438 | Alphacarmovirus |
| Oat chlorotic stunt virus | OCSV | NC003633 | Avenavirus |
| Olive latent virus 1 | OLV1 | X85989 | Alphanecrovirus |
| Olive mild mosaic virus | OMMV | AY616760 | Alphanecrovirus |
| Opium poppy mosaic virus | OPMV | EU151723 | Umbravirus |
| Opuntia umbra-like virus | OULV | MH579715 | Unclassified |
| Panicum mosaic virus | PMV | NC002598 | Panicovirus |
| Papaya meleira virus 2 | PMeV2 | KT921785 | Unclassified |
| Papaya virus Q | PpVQ | MT113180 | Unclassified |
| Patrinia mild mottle virus | PMMoV | MH922775 | Unclassified |
| Pea enation mosaic virus 2 | PEMV2 | U03563 | Umbravirus |
| Pea stem necrosis virus | PSNV | AB086951 | Gammacarmovirus |
| Pear latent virus | PeLV | AY100482 | Tombusvirus |
| Pelargonium chlorotic ring pattern virus | PCRPV | NC005985 | Pelarspovirus |
| Pelargonium flower break virus | PFBV | AJ514833 | Alphacarmovirus |
| Pelargonium leaf curl virus | PLCV | KU187189 | Tombusvirus |
| Pelargonium line pattern virus | PLPV | NC007017 | Pelarspovirus |
| Pelargonium necrotic spot virus | PNSV | AJ607402 | Tombusvirus |
| Pelargonium ringspot virus | PelRSV | NC026240 | Pelarspovirus |
| Pothos latent virus | PoLV | NC000939 | Aureusvirus |
| Potato necrosis virus | PNV | KP901095 | Alphanecrovirus |
| Red clover necrotic mosaic virus | RCNMV | J04357 | Dianthovirus |
| Red clover umbravirus | PCUV | MG596234 | Unclassified |
| Rosa rugosa leaf distortion virus | RrLDV | NC020415 | Pelarspovirus |
| Saguaro cactus virus | SgCV | U72332 | Alphacarmovirus |
| Soybean yellow mottle mosaic virus | SYMMV | FJ457015 | Gammacarmovirus |
| Sugarcane umbra-like virus | SULV | MN868593 | Unclassified |
| Sweet clover necrotic mosaic virus | SCNMV | NC003806 | Dianthovirus |
| Thin paspalum asymptomatic virus | TPAV | NC021705 | Panicovirus |
| Tobacco bushy top disease-associated RNA | TBTDaRNA | EF529625 | Tombusvirus like associated RNA |
| Tobacco bushy top virus | TBTV | NC004366 | Umbravirus |
| Tobacco necrosis virus A | TNV-A | M33002 | Alphanecrovirus |
| Tobacco necrosis virus D | TNV-D | U62546 | Betanecrovirus |
| Tomato bushy stunt virus | TBSV | M21958 | Tombusvirus |
| Trailing lespedeza virus 1 | TLV 1 | HM640935 | Unclassified |
| Turnip crinkle virus | TCV | M22445 | Betacarmovirus |
| Wild carrot mottle virus | WCMoV | LT615232 | Unclassified |
| Yam spherical virus | YSV | NC022895 | Aureusvirus |
